# Hypothalamic TLR2 triggers sickness behavior via a microglia-neuronal axis

**DOI:** 10.1038/srep29424

**Published:** 2016-07-12

**Authors:** Sungho Jin, Jae Geun Kim, Jeong Woo Park, Marco Koch, Tamas L. Horvath, Byung Ju Lee

**Affiliations:** 1 Department of Biological Sciences, University of Ulsan, Ulsan, 680-749, Republic of Korea; 2 Division of Life Sciences, College of Life Sciences and Bioengineering, Incheon National University, Incheon, 406-772, Republic of Korea; 3 Program in Integrative Cell Signaling and Neurobiology of Metabolism, Section of Comparative Medicine, Yale University School of Medicine, New Haven, CT 06520, USA; 4 Institute of Anatomy, University of Leipzig, 04103 Leipzig, Germany

## Abstract

Various pathophysiologic mechanisms leading to sickness behaviors have been proposed. For example, an inflammatory process in the hypothalamus has been implicated, but the signaling modalities that involve inflammatory mechanisms and neuronal circuit functions are ill-defined. Here, we show that toll-like receptor 2 (TLR2) activation by intracerebroventricular injection of its ligand, Pam3CSK4, triggered hypothalamic inflammation and activation of arcuate nucleus microglia, resulting in altered input organization and increased activity of proopiomelanocortin (POMC) neurons. These animals developed sickness behavior symptoms, including anorexia, hypoactivity, and hyperthermia. Antagonists of nuclear factor kappa B (NF-κB), cyclooxygenase pathway and melanocortin receptors 3/4 reversed the anorexia and body weight loss induced by TLR2 activation. These results unmask an important role of TLR2 in the development of sickness behaviors via stimulation of hypothalamic microglia to promote POMC neuronal activation in association with hypothalamic inflammation.

A growing body of evidence indicates that an inflammatory process in the hypothalamus is one of the major causes of dysfunctions in energy metabolism. In particular, previous studies have focused on events of chronic inflammation, which is an important pathologic element leading to metabolic syndromes^1^,^2^. However, the significance of acute hypothalamic inflammation in sickness behaviors has been relatively ignored even though it is closely correlated with human diseases such as infection and cachexia^3^. Sickness responses are coordinated sets of adaptive behavioral changes including loss of appetite, hypoactivity, and hyperthermia during the course of human diseases such as cancer, acquired immune deficiency syndrome (AIDS), tuberculosis, and renal failure^4^. Although hypothalamic inflammation has been recognized as a prominent component of sickness responses^5^,^6^, the pathophysiologic mechanisms of sickness symptoms are poorly understood.

Toll-like receptors (TLRs) are key components of the innate immune system through their role in the recognition of a variety of pathogens and inflammatory signals^7^. Recently, it has been proposed that perturbation of metabolic controls is closely associated with the function of TLRs as inflammatory modulators^8^,^9^,^10^. In the development of diet- or ageing-induced obesity, the function of TLRs in the neural cells is thought to be as important as their impact on peripheral organs^11^,^12^. Nevertheless, it remains unclear whether TLRs participate in the development of sickness behaviors governed by hypothalamic neural circuits. Therefore, in this study, we interrogated the role of hypothalamic TLR2 in the development of sickness behaviors caused by acute hypothalamic inflammation.

We found that intracerebroventricular (icv) injection of the TLR2 ligand Pam3CSK4 led to sickness responses including anorexia, hypoactivity, and hyperthermia through stimulation of inflammatory processes in the hypothalamus. In addition, we observed that TLR2 caused microglia activation in the hypothalamic arcuate nucleus (Arc). Since the hypothalamic melanocortin pathway is involved in inflammation-induced negative energy balance^13^,^14^, we further investigated the interrelationship between microglia activation and the melanocortin pathway in the development of sickness behaviors induced by TLR2 activation.

## Results

### Central TLR2 activation leads to sickness behaviors

**Figure 1:**
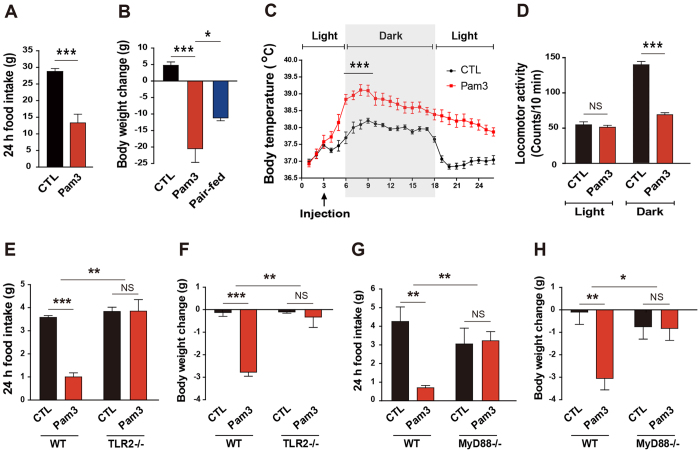
Centrally activated TLR2 leads to sickness responses. (**A**,**B**) Changes in food intake (**A**) and body weight (**B**) were measured in rats for 24 h after icv injection of vehicle (CTL) or Pam3CSK4 (Pam3). The pair-fed group was provided with the average amount of food consumed by Pam3-injected rats for 24 h (n = 6–8 rats/group; *P < 0.05, ***P < 0.0001 by unpaired two-tailed Student’s t-tests). (**C**,**D**) Body temperature (**C**) and locomotor activity (**D**) were monitored in rats that were treated icv with vehicle or Pam3 using the Vital View system (n = 6–8 rats/group; *P < 0.05, ***P < 0.0001 by unpaired two-tailed Student’s t-tests; NS, not significant). (**E**,**F**) Food intake (**E**) and body weight (**F**) were determined in TLR2 KO mice for 24 h after icv injection of vehicle or Pam3 (n = 3–4 mice/group, **P < 0.01, ***P < 0.001 by two-way ANOVA; NS, not significant). (**G**,**H**) Food intake (**G**) and body weight (**H**) were measured in MyD88 KO mice for 24 h after icv injection of vehicle or Pam3 (n = 3–5 mice/group; *P < 0.05, **P < 0.01, ***P < 0.001 by two-way ANOVA; NS, not significant). (**A**–**D**) Data from rat models. All data are presented as mean ± s.e.m.

Although lipopolysaccharide (LPS), an endotoxin, is a well-known inducer for the sickness behaviors, it stimulates TLR4 as well as TLR2 in the brain cells such as microglia. To more directly clarify the role of TLR2 in central nervous system (CNS)-controlled sickness behaviors, we centrally administered Pam3CSK4, a synthetic agonist of TLR2, and examined sickness behaviors including anorexia, hypoactivity, and hyperthermia. Intracerebroventricular (icv) injection of Pam3CSK4 resulted in a decrease in food intake (Fig. 1A) and body weight (Fig. 1B) of 2-month-old male rats. Notably, the weight loss observed in the Pam3CSK4-treated group was greater than that in the pair-fed group given the same amount of food as the Pam3CSK4-treated group (Fig. 1B). In line with this observation, we observed that central activation of TLR2 increased core body temperature (Fig. 1C). On the other hand, the locomotive activity in Pam3CSK4-treated rats was significantly lower than that of vehicle-treated rats during the dark period (Fig. 1D). To further validate whether activation of TLR2 is responsible for the Pam3CSK4-induced sickness behaviors, we determined changes in food intake and body weight in TLR2 knockout (KO) mice after icv administration of Pam3CSK4. The anorexia and weight loss observed in Pam3CSK4-treated wild-type mice were completely rescued in TLR2 KO mice (Fig. 1E,F).

Since myeloid differentiation factor 88 (MyD88) is an adapter protein for intracellular signaling of TLRs^15^, we further tested the behavioral phenotypes seen in Pam3CSK4-treated mice using MyD88 KO mice. Consistent with the phenotypes of TLR2 KO mice, MyD88 deficiency dramatically reduced the Pam3CSK4-induced anorexia (Fig. 1G) and body weight loss (Fig. 1H). These findings suggest that activation of TLR2 signals induces sickness responses.

### TLR2 signaling triggers microglial activation in the hypothalamic arcuate nucleus

**Figure 2:**
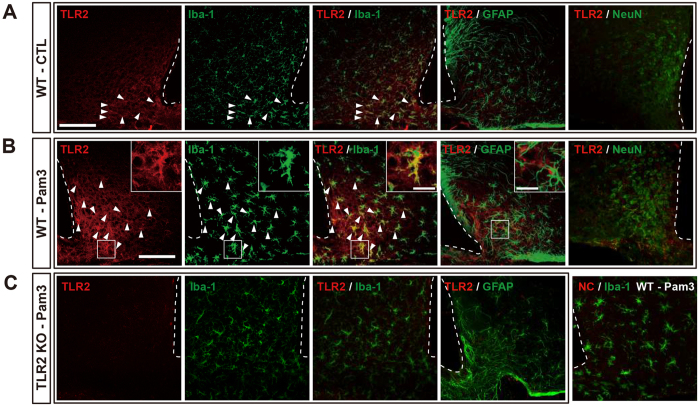
TLR2 signaling-induced morphological change of microglia in the mouse hypothalamic Arc. Central administration of Pam3CSK4 (Pam3) resulted in microglia-specific induction of TLR2. (**A**) TLR2 immunosignals (red) were co-localized with signals of Iba-1 (green), a marker for microglia, but not with signals of GFAP (green), a marker for astrocytes, in the arcuate nucleus of the saline-injected mouse brain. (**B**) Central administration of Pam3 caused exclusive induction of TLR2 in the Iba-1-positive cells. Arrow heads: double-labeled TLR2 and Iba-1-positive cells. (**C**) Pam3CSK4 (Pam3)-induced activation of microglia and TLR2 expression were absent in the TLR2 KO mice. NC = negative control for TLR2 (without primary antibody). Scale bar = 100 μm (20 μm for higher magnification view in insets).

To identify the cellular targets of TLR2 action in the development of sickness behaviors, we evaluated the patterns of TLR2 localization in mouse hypothalamic cells that were immunopositive for either ionized calcium binding adaptor molecule-1 (Iba-1), a microglia marker, glial fibrillary acidic protein (GFAP), an astrocyte marker or neuronal nuclear antigen (NeuN), a neuronal marker. Under normal conditions, relatively weak TLR2-positive signals were found in microglial cells, whereas the TLR2-positive cells did not express GFAP or NeuN in the Arc of the mouse hypothalamus (Fig. 2A). Central administration of Pam3CSK4 induced TLR2 expression in the Iba-1-positive microglia but not in the GFAP-positive astrocytes and NeuN-positive neuronal cells (Fig. 2B) and strongly activated microglia in the hypothalamic ARC (Supplementary Fig. S1). However, the *TLR2* KO mice revealed neither positive TLR2 signal nor the activation of microglia induced by Pam3CSK4 (Fig. 2C, Supplementary Fig. S1). We also found that central administration of Pam3CSK4 did not alter the morphology of Iba-1-positive cells in the mouse hippocampus and cortex (Supplementary Fig. S2A,B). In the area postrema (AP) and organum vasculosum of the lamina terminalis (OVLT) where are regions adjacent to the ventricle and thus easily accessible to the cerebrospinal fluid (CSF), microglia revealed a slightly activated morphology after icv Pam3CSK4 administration (Supplementary Fig. S2C,D). However, the change was not so much dramatic as observed in the hypothalamic Arc, though reason for this discrepancy of the Pam3CSK4 effectiveness is unclear. These data indicate that icv administration of Pam3CSK4 is effective tool for study of the microglial activation in circumventricular organs (CVOs), especially in the hypothalamic Arc.

### Hypothalamic microglia play an essential role in the development of anorexia and body weight loss

**Figure 3:**
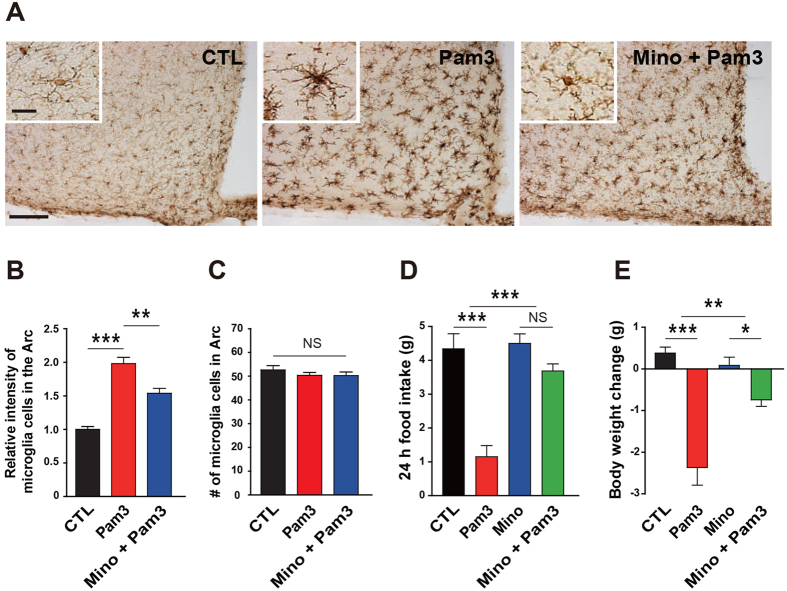
Microglia activation is responsible for the anorexia and body weight loss induced by TLR2 in the mouse hypothalamic Arc. (**A**) Representative images (3–4 mice analyzed) show immunosignals of Iba-1 in the hypothalamic Arc. Scale bar = 100 μm (20 μm for higher magnification view in inset). (**B**,**C**) Increased intensity of Iba-1 signals in the Arc (**B**) of icv Pam3CSK4 (Pam3)-injected mice was significantly attenuated by preadministration of minocycline (Mino), a bacteriostatic antibiotic that effectively inhibits microglia activation. There was no difference in the number of microglia (**C**) among different treatment groups (CTL, n = 8 section/4 mice; Pam3, n = 6 section/3 mice; Mino + Pam3, n = 8 section/4 mice; ** P < 0.01, ***P < 0.0001 by unpaired two-tailed Student’s t-tests; NS, not significant). (**D**,**E**) Pam3-induced anorexia (**D**) and weight loss (**E**) were significantly mitigated by administration of Mino for 3 consecutive days prior to icv Pam3 injection. (CTL, n = 5 mice; Pam3, n = 6; Mino, n = 7; Mino + Pam3, n = 7; *P < 0.05, **P < 0.01, ***P < 0.001 by two-way ANOVA; NS, not significant). All data are presented as mean ± s.e.m.

In line with these previous anatomical findings showing microglia-specific induction of TLR2 by Pam3CSK4, we found that Pam3CSK4-induced TLR2 stimulation resulted in an elevated intensity of Iba-1 signals as well as morphologic changes reflecting microglia activation in the Arc (Fig. 3A,B).

To verify the impact of Pam3CSK4-induced microglia activation on the changes in feeding behavior, we administered icv minocycline, which is commonly used to inhibit microglia activation, prior to the Pam3CSK4 injection. In support of data validating the suppressive effect of minocycline on the hypothalamic microglia activation (Fig. 3A–C), minocycline almost completely abolished the Pam3CSK4-induced anorexia and subsequent body weight loss (Fig. 3D,E). To further confirm role of microglia in the TLR2-induced changes in feeding behavior, we selectively decreased microglia number with icv administration of liposome-encapsulated clodronate (Clo-lip), which effectively ablated macrophages in various organs^16^ and microglia in the CNS^17^, before administration of Pam3CSK4. Clo-lip effectively decreased Iba-1-positive microglia in the hypothalamic Arc (Supplementary Fig. S3A,B), and significantly mitigated the Pam3CSK4-induced anorexia (Supplementary Fig. S3C) and body weight loss (Supplementary Fig. S3D). These findings suggest that hypothalamic microglia are important cellular components in the development of the anorexia and body weight loss caused by TLR2 activation.

### Hypothalamic inflammatory responses are responsible for the TLR2-induced sickness responses

To determine whether TLR2-activated sickness responses are associated with hypothalamic inflammation, we evaluated mRNA levels of proinflammatory cytokines and prostaglandin-synthesizing enzymes in the hypothalamus of vehicle-treated mice and mice receiving Pam3CSK4 using real-time qPCR analysis. Icv administered Pam3CSK4 significantly enhanced TLR2 mRNA expression and stimulated mRNA expression of proinflammatory cytokines such as tumor necrosis factor-alpha (TNF-α) and interleukin-1 beta (IL-1β) as well as prostaglandin-synthesizing enzymes including cyclooxygenase 2 (COX2) and microsomal prostaglandin E synthase-1 (mPGES-1) in the hypothalamus (Supplementary Fig. S4). However, PamCSK4 did not induce significant changes in mRNA expression of IL-1β and TNF-α in the cortex samples (Supplementary Fig. S4A). In addition, Pam3CSK4-induced elevated expression of genes involved in inflammatory processes was completely absent in TLR2 KO mice (Supplementary Fig. S4B) and MyD88 KO mice (Supplementary Fig. S4C).

**Figure 4:**
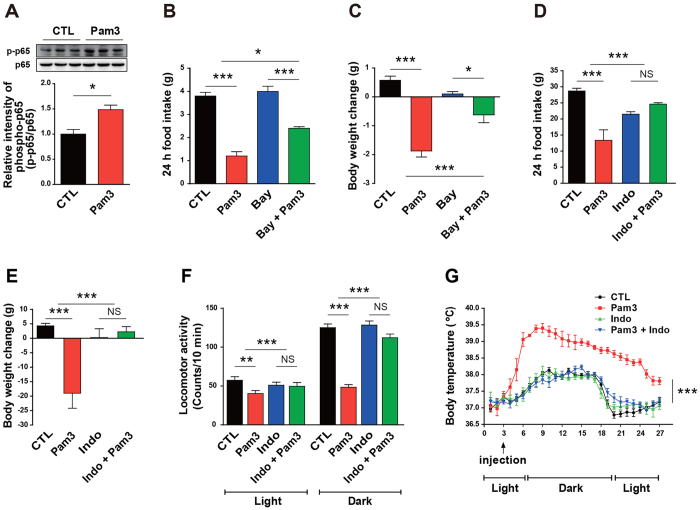
Involvement of NF-κB and COX pathway in the TLR2-associated sickness behaviors. Mice received an NF-κB inhibitor, Bay 11-7085 (Bay) or rats received a COX inhibitor, indomethacin (Indo), 1 h prior to icv injection of Pam3CSK4 (Pam3), a synthetic TLR2 ligand, and changes in sickness responses were monitored. (**A**) Hypothalamic content of phosphorylated p65, a subunit of NF-κB, was determined in 8-week-old male rats after icv injection with either vehicle (CTL) or Pam3 (n = 3 rats/group; *P < 0.05 by unpaired two-tailed Student’s t-tests). (**B**,**C**) Food intake (**B**) and body weight (**C**) were assessed in mice that were treated icv with either vehicle or Bay 1 h prior to Pam3 injection (CTL, n = 4 mice; Pam3, n = 8; Bay, n = 7; Bay + Pam3, n = 4; *P < 0.05, ***P < 0.001 by two-way ANOVA). (**D**–**G**) Changes in food intake (**D**), body weight (**E**), locomotor activity (**F**), and body temperature (**G**) were monitored in rats that were intraperitoneally injected with either vehicle or Indomethacin 1 h prior to administration of Pam3 (n = 4–6 rats/group; **P < 0.01, ***P < 0.001 by two-way ANOVA; NS, not significant). (**A**,**D**–**G**) Data from rat models. (**B**,**C**) Mouse models. All data are presented as mean ± s.e.m.

To identify a mechanistic pathway linked to the sickness responses induced by central TLR2 activation, we further interrogated downstream components of the hypothalamic TLR2 signaling cascade. First, we observed that central administration of Pam3CSK4 stimulated phosphorylation of p65, a subunit of nuclear factor kappa B (NF-κB), in the rat hypothalamus (Fig. 4A). Bay 11–7085, a synthetic NF-κB inhibitor, was injected icv into the mouse brain and its effect on the Pam3CSK4-induced sickness responses was examined. Bay 11-7085-mediated blockade of NF-κB signaling mitigated Pam3CSK4-induced anorexia (Fig. 4B) and weight loss (Fig. 4C), which suggests an important role of NF-κB in the Pam3CSK4-induced sickness behaviors. Since COX, the rate-limiting enzyme for synthesis of PGE2, plays an important role in the development of sickness responses during inflammatory states^18^, and moreover, sickness behaviors and plasma PGE2 concentration caused by endotoxin-induced systemic inflammation were reversed by indomethacin^19^,^20^, a COX inhibitor, we then determined the effect of COX pathway on the sickness responses using indomethacin. Rats were intraperitoneally injected with indomethacin 30 min before icv injection of Pam3CSK4 and metabolic profiles were monitored. Indomethacin-induced inhibition of COX synthesis resulted in a complete disappearance of the Pam3CSK4-induced sickness responses during the observation period (Fig. 4D–G). These results show that activation of TLR2 signaling in the brain triggers sickness behaviors, at least in part, via activation of the NF-κB and COX pathway.

### TLR2-induced microglia activation changes synaptic input organization of proopiomelanocortin (POMC) neurons

**Figure 5:**
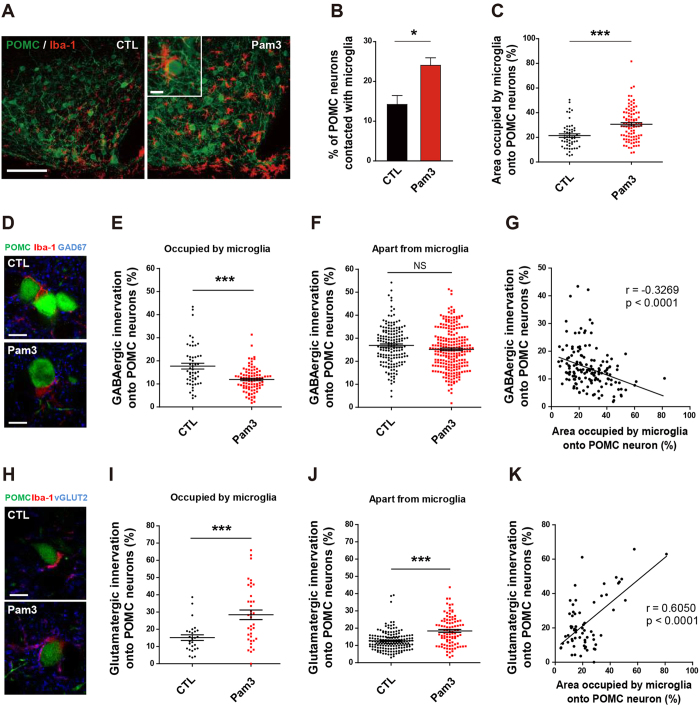
TLR2-induced microglia activation alters synaptic inputs of POMC neurons. (**A**) Images of POMC-GFP (green) and Iba1-positive microglia (red) in the arcuate nucleus after saline (CTL) or Pam3CSK4 (Pam3) injection. Scale bar = 100 μm (10 μm in inset). (**B**) Percent of POMC cells in contact with microglial cells in CTL and Pam3-injected mice (n = 5 section/3 mice/group; *P < 0.05 by unpaired two-tailed Student’s t-tests). (**C**) Percentage of microglia area of POMC neurons in Pam3- and saline-injected mice (CTL, n = 53 cells/4 mice; Pam3, n = 95 cells/7 mice; ***P < 0.0001 by unpaired two-tailed Student’s t-tests). (**D**) Triple labeling of POMC neurons (green), microglia (red), and GAD 67 (blue). Scale bar = 10 μm. (**E**,**F**) Pam3 decreased percentage of GABAergic synapses on the surface of POMC soma in contact with activated microglia (**E**), CTL, n = 53 cells/4 mice; Pam3, n = 95 cells/7 mice; ***P < 0.0001 by unpaired two-tailed Student’s t-tests) but not on POMC cells separate from microglia (**F**), CTL, n = 166 cells/4 mice; Pam3, n = 243 cells/7 mice; NS). (**G**) Scatter plots show a negative correlation between the GABAergic innervation and the area occupied by microglia onto POMC neurons. (n = 148 cells in contact with microglia/11 mice; r = −0.3269, P < 0.0001 by Pearson correlation coefficient analysis with a two-tailed test). (**H**) Confocal micrographs of POMC neurons (green), microglia (red), and vGlut2 (blue). Scale bar = 10 μm. (**I**,**J**) Glutamatergic synapses on POMC soma in contact with microglia (**I**), CTL, n = 27 cells/2 mice; Pam3, n = 38 cells/2 mice; ***P < 0.0001 by unpaired two-tailed Student’s t-tests) or separated from microglia (**J**) CTL, n = 161 cells/2 mice; Pam3, n = 95 cells/2 mice; ***P < 0.0001 by unpaired two-tailed Student’s t-tests). (**K**) Scatter plots show positive correlation between the glutamatergic innervation and the area occupied by microglia onto POMC neurons. (n = 65 cells in contact with microglia/4 mice; r = 0.6050, P < 0.0001 by Pearson correlation coefficient analysis with a two-tailed test). All data are presented as mean ± s.e.m.

We recently reported that functional contacts between astrocytic processes and the POMC neurons are linked to synaptic plasticity of the hypothalamic neuronal circuits regulating feeding^21^. Considering the anatomic findings suggesting that TLR2 signaling induced specific and dominant activation of microglia in the Arc, we tested whether this spatial microglia activation might affect the pattern of interaction between microglia and POMC cells in the Arc, which are essential for the control of feeding^22^. Icv administration of Pam3CSK4 significantly increased direct contacts between POMC cells and Iba-1-positive microglia relative to control values (Fig. 5A,B). In addition, we found that central administration of Pam3CSK4 induces an elevation of microglia occupation onto the surface of POMC soma (Fig. 5C). On the basis of these data and our previous findings, we assessed the effect of Pam3CSK4-induced morphological changes of microglia on input organization of POMC neurons using confocal microscopy. We analyzed the synaptic input organization onto the POMC neurons using POMC-green fluorescent protein (GFP) mice injected with saline or Pam3CSK4. Sections containing GFP-labeled POMC cells were processed for double immunolabeling with anti-glutamic acid decarboxylase 67 (GAD67) antibodies to identify inhibitory GABAergic presynaptic terminals and anti-Iba-1 antibodies to detect microglia. Analyses of triple labeled Arc sections revealed that administration of Pam3CSK4 significantly reduced the rate of GABAergic contacts onto the surface of POMC soma occupied by microglia (Fig. 5D,E), but not onto the POMC soma separate from microglia (Fig. 5F). Calculated data sets clearly showed an effect of activated microglia on the contacted POMC cells but not on those separate from microglia. We further assessed the patterns of GABAergic innervation correlated with area occupied by microglia onto the surface of POMC soma. Interestingly, the rate of GABAergic innervation onto the surface of POMC soma negatively correlated with the change in area occupied by microglia onto the surface of POMC soma (Fig. 5G). These results are coincident with previous studies showing that activated microglia can reduce the inhibitory synapses from the neuronal cell body^23^,^24^. Coronal sections containing GFP-labeled POMC cells were incubated with anti-vesicular glutamate transporter 2 (vGLUT2) antibody to identify excitatory glutamatergic presynaptic terminals in combination with immunostaining of Iba-1. Unlike inhibitory GABAergic innervation, administration of Pam3CSK4 increased bouton signals of vGLUT2 onto the surface of POMC soma that were either occupied or separated from microglia (Fig. 5H–J). We also observed that administration of Pam3CSK4 significantly increased the rate of glutamatergic innervation onto the surface of POMC soma positively correlated with the change in area occupied by microglia onto the surface of POMC soma (Fig. 5K). Taken together, these data suggest that activated microglia control synaptic input organization onto the POMC neurons.

### POMC neurons mediate anorexia and body weight loss triggered by TLR2-induced microglia activation

**Figure 6:**
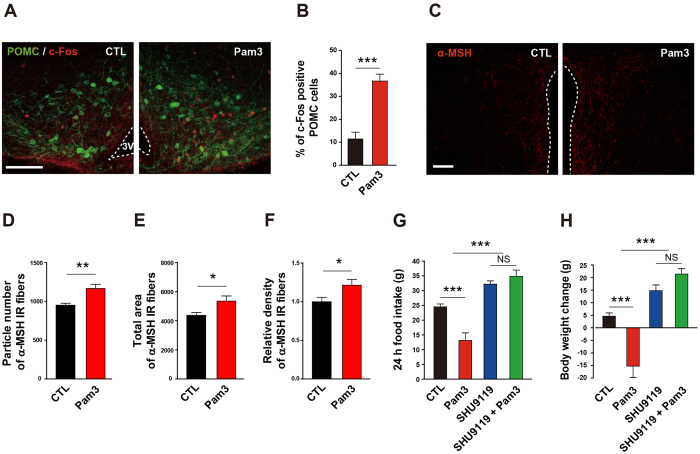
Involvement of melanocortin pathway in the TLR2-induced anorexia and body weight loss. (**A**,**B**) POMC neuronal activity was determined by the change in c-Fos immunoreactivity in the arcuate nucleus of mice that were icv treated with Pam3CSK4 (Pam3) 1 h before sacrifice. Representative photographs (**A**) and calculated graphs (**B**) reveal an increase in the number of c-Fos positive POMC cells induced by Pam3 (CTL, n = 4 section/2 mice; Pam3, n = 6 section/3 mice; ***P < 0.0001 by unpaired two-tailed Student’s t-tests). Scale bar = 100 μm. (**C**–**F**) Effect of Pam3 on POMC neuronal terminals of the hypothalamic paraventricular nucleus (PVN) was determined by α-melanocyte stimulating hormone (α-MSH) immunoreactivity. Representative images (**C**) show an increase in α-MSH immunosignals in the PVN after Pam3 injection. Scale bar = 100 μm. Icv administration of Pam3 led an increase in particle number (**D**), total area (**E**), and relative density (**F**) of α-MSH fiber signals (CTL, n = 12 sections/6 mice; Pam3, n = 16 sections/8 mice; *P < 0.05, **P < 0.01 by unpaired two-tailed Student’s t-tests). (**G**,**H**) Effect of antagonizing melanocortin 3 and 4 (MC3/4) receptors on Pam3-induced sickness behaviors. Changes in food intake and body weight were monitored in rats after icv treatment with SHU9119, an MC3/4 receptor antagonist. The antagonist completely abolished the inhibitory effect of Pam3 on food intake (**G**) and body weight (**H**) (n = 4–5 rats/group; ***P < 0.001 by two-way ANOVA; NS, not significant). (**G**,**H**) Data from rat model. All data are presented as mean ± s.e.m.

It has previously been reported that the melanocortin pathway participates in the control of negative energy balance and is involved in disease-related metabolic syndromes such as anorexia and cachexia^14^,^25^,^26^,^27^. We observed that Pam3CSK4 stimulated neuronal activity in the Arc, as determined by locally increased immunoreactivity for c-Fos, a marker of neuronal activity (Supplementary Fig. S5). In particular, administration of Pam3CSK4 promoted POMC neuronal activity as assessed by counting c-Fos-positive POMC cells (Fig. 6A,B). The POMC gene encodes α-melanocyte-stimulating hormone (α-MSH), which targets the paraventricular nucleus (PVN) where efferent POMC neurons are thought to trigger satiety signals^28^. Thus, we analyzed the patterns of PVN α-MSH fibers. Central administration of Pam3CSK4 resulted in an increase in α-MSH fibers in the hypothalamic PVN (Fig. 6C–F), further confirming that the melanocortin pathway may mediate central TLR2-induced anorexia and body weight loss. In line with this, we observed that SHU9119, a synthetic antagonist of melanocortin 3 and 4 (MC3/4) receptors, completely reversed the anorexia (Fig. 6G) and weight loss (Fig. 6H) seen in Pam3CSK4-treated rats.

Taken together, the aforementioned findings unmasked a functional interaction between microglia and POMC neurons in sickness behaviors induced by central TLR2 activation (Supplementary Fig. S6).

## Discussion

The present study highlights a prominent role of hypothalamic TLR2 in the development of sickness behaviors caused by acute hypothalamic inflammation and links the functional interaction between microglia and POMC neurons with these pathologic processes. These findings reveal a previously underappreciated role of microglia in the short-term regulation of energy metabolism and support evidence that microglia act as a primary conductor of synaptic plasticity of the hypothalamic neurocircuitry for the control of energy balance.

Toll-like receptors (TLRs) have been identified as pattern recognition receptors (PRRs) for the recognition of pathogen-associated molecular patterns (PAMPs) leading to innate immune reaction to invading pathogen^29^. TLR signaling is mediated mainly by the adaptor molecule MyD88. Although all TLRs except TLR3 trigger the MyD88-dependent signaling pathway, stimulation of TLR4 by endotoxin such as LPS can also use MyD88-independent pathway^30^. However, it has been well known that TLR2 heterodimers (with TLR1 or TLR6) predominantly require the MyD88-dependent signaling pathway for their action^31^. Bacterial endotoxin and/or endotoxin-induced inflammatory cytokines originated from the peripheral tissues can cause brain inflammation. However, in the brain, there is blood-brain barrier (BBB) that is a selective permeability barrier to protect the CNS from harmful factors^32^,^33^. Nevertheless, circulating endotoxin can enter the brain by changing permeability of BBB and/or through the CVOs, such as the OVLT, the subfornical organ and the median eminence, which lack BBB^34^,^35^.

TLR signaling induces NF-κB activation in myeloid cells through degradation of the NF-κB inhibitor nuclear factor of kappa light polypeptide gene enhancer in B-cells inhibitor, alpha (IκBα), resulting in nuclear translocation of NF-κB for transcriptional regulation of target genes that regulate inflammation. Recent reports have shown that NF-κB activation in hypothalamic cells leads to acute negative energy balance^26^,^36^ as well as chronic inflammation induced by ageing and overnutrition^2^,^37^,^38^. In the present study, we showed that inhibition of NF-κB activity by icv administration of a synthetic inhibitor rescued the animals from sickness behaviors caused by TLR2 activation, suggesting that NF-κB mediates TLR2-induced sickness responses in the brain. Previous *in vivo* studies have documented that selective pharmacologic or genetic blockade of COX2 attenuates sickness behaviors in response to systemic inflammation induced by lipopolysaccharide (LPS)^18^,^39^. Consistent with these reports, we found that the COX inhibitor, indomethacin, effectively ameliorated TLR2-induced sickness behaviors. Collectively, our findings provide evidence supporting the hypothesis that TLR2 is a molecular component in the development of inflammation-induced sickness behaviors in the hypothalamus that transfers signals to the NF-κB and COX pathways.

We next addressed cells in which TLR2 functions in the development of sickness behaviors. Previous studies have reported that TLR2 is stimulated in microglia after brain damage^40^,^41^. Indeed, we observed that TLR2 was activated in microglia of the hypothalamic Arc by a synthetic agonist, thereby resulting in microglia activation. However, intriguingly, there was no specific TLR2 expression in the astrocytes and neurons of Arc in either the normal or TLR2-activated state. Paradoxically, a recent study localized TLR2 expression to the POMC neurons, but not to astrocytes or microglia, in the hypothalamic Arc of untreated middle-aged mice^12^. These different expression patterns of TLR2 between the earlier study and ours may be due in part to different age effects (middle vs. young age), and/or our specific experimental model in which brain TLR2 was directly stimulated by icv injection of its ligand, leading to microglia-specific induction of TLR2 in the hypothalamic Arc.

Since cells in the CNS dynamically communicate with each other, pathophysiological changes governed by CNS could not be simply explained by a single cellular cause. It has been known that activated microglia affect astrogliosis^42^,^43^ and oligodendrogenesis^44^,^45^ under pathological conditions. Therefore, it is possible that the microglia activation induced by TLR2 signaling alters status of other cells such as astrocytes and oligodendrocytes.

Recently, much effort has been paid to unmask the role of the melanocortin pathway in the development of inflammation-related abnormalities of energy balance and disease-related eating disorders such as anorexia and cachexia^13^,^14^. It has also been reported that overnutrition-induced hypothalamic gliosis accompanied by hypothalamic inflammation led to injury of POMC neurons, a major component of the melanocortin system^1^. We showed that physical contacts between microglia and POMC neurons increased in the hypothalamic Arc after icv administration of TLR2 ligand. Moreover, POMC neuronal activity was increased in this condition. In support of these findings, we successfully rescued mice from TLR2 ligand-induced anorexia by icv administration of an antagonist against MC3/4 receptors. These observations strengthen the notion that microglia are primary controllers in the regulation of appetite through the melanocortin pathway.

Further studies are required to clarify the underlying mechanisms by which microglia directly (or indirectly) control the POMC neurons. Until now, a role of microglia as a representative of the CNS immune system has been favored. In particular, previous reports provided evidence for a relationship between inflammatory signals and status of the POMC neurons^13^. Therefore, to better understand the cellular mechanisms of microglia-regulated POMC neuronal activity it is necessary to clarify whether microglia-derived inflammatory cytokines and trophic factors affect the activity of the hypothalamic neurocircuitry in normal and/or pathogenic conditions. Since all types of brain cells, including neuronal and non-neuronal cells, communicate through physical interactions as well as paracrine factors^46^,^47^, we suggest that the complete interpretation of brain functions cannot be reliably assessed by contemporary ways of neuronal circuit analysis.

Previous studies have highlighted that hypothalamic non-neuronal cells are also important for the maintenance of neuronal energy supplies and participate in synaptic plasticity of hypothalamic neurons. In addition, we recently identified that astrocytic processes induced in response to altered leptin receptor signaling play a role of gatekeeper to orchestrate the pattern of synaptic inputs, which drive the activity of POMC and NPY/AgRP neurons^21^. Microglia also act as phagocytes to clean up CNS debris and eliminate synapses^48^,^49^. Although several studies by us and others have proposed an active role of glial cells in the synaptic plasticity of neuronal circuits, less is known about microglial regulation of synaptic input organization that occurs in the hypothalamus during different metabolic states. In this regard, we propose that microglia might be upstream controllers in the synaptic plasticity of hypothalamic neurons, beyond their classic functions. In line with this, we observed that activation of central TLR2 resulted in microglia activation in the Arc that resulted in a subsequent increase in POMC cells occupied by the activated microglia. We showed that reactive microglia decrease inhibitory GABAergic inputs and increase excitatory glutamatergic inputs onto POMC neurons while promoting POMC neuronal activity. These findings lead to the suggestion that microglia are orchestrators in controlling synaptic input organization onto the hypothalamic POMC neurons.

This study also raised an open question: How do activated microglia selectively control inhibitory and excitatory synaptic inputs onto the POMC neurons? Intriguingly, our previous report showed that reduced astrocyte coverage onto POMC neurons increased inhibitory synaptic inputs but not excitatory synaptic inputs^28^. In addition, another research group also reported that LPS-induced activated microglia selectively displaced synapses from hippocampal neurons^24^. Therefore, it might be valuable to further interrogate mechanisms underlying the function of glial processes in controlling the balance of inhibitory and excitatory synapse inputs onto the hypothalamic neurons to reveal homeostatic regulations according to changes in metabolic status.

Collectively, the current study identified an active role of hypothalamic TLR2 in promotion of sickness behaviors induced by hypothalamic inflammation. Our results indicate that physical interactions between microglia and melanocortin cells are important component in the development of sickness behaviors.

## Methods

### Animals and dietary protocols

Adult male 8-week-old rats and mice were used in the pharmacologic studies. Animals were fed standard chow (Feedlab, Gyeonggi-Do, Korea) ad libitum, unless otherwise stated. All animals were maintained in a temperature and humidity controlled room with a 12 h-12 h light-dark cycle, with light on from 6:00 a.m. to 6:00 p.m. Mice deficient in toll-like receptor 2 (TLR2-/-) or myeloid differentiation primary-response protein 88 (MyD88-/-) were maintained under specific pathogen-free conditions (University of Ulsan, Ulsan, Korea). Transgenic mice expressing green fluorescent protein (GFP) in the proopiomelanocortin (POMC) neurons (POMC-GFP mice, #008322, The Jackson Laboratories, Bar Harbor, ME, USA) were used for immunohistochemical analysis. All animal care and experimental procedures were performed in accordance with a protocol approved by the Institutional Animal Care and Use Committee (IACUC) at the University of Ulsan (Permission number: UOU-2013-002, UOU-14-030 and UOU-2015-008).

### Intracerebroventricular (icv) cannulation

Mice and rats were anesthetized with an intraperitoneal (ip) injection of tribromoethanol (250 mg/kg, Sigma-Aldrich, St. Louis, MO, USA) and placed in a stereotaxic apparatus (Stoelting, Wood Dale, IL, USA). For mice, the cannula (26 gauge) was implanted into the right lateral ventricle (coordinates of 0.1 mm lateral, 0.03 mm posterior and 2.4 mm ventral to the bregma) and secured to the skull with dental cement. For rats, a polyethylene cannula (o.d. 1.05 mm, i.d. 0.35 mm) was implanted into the lateral ventricle. Coordinates were +0.16 mm (lateral), - 0.1 mm (posterior), +3.6 mm (ventral) from bregma. Animals were kept warm until they recovered from the anesthesia and then placed in individual cages. After surgery, a recovery period of 7 days was allowed before starting experiments. Test materials were injected through the cannula.

### Drug administration

All animals were randomly assigned to different experimental groups. For icv injection of Pam3CSK4 (Pam3, a TLR2 agonist; EMC microcollection, Tübingen, Germany), Pam3 (200 ng/2 μl for mouse, 1.0 μg/3 μl for rat) was slowly infused into the lateral ventricle at a rate of 2 μl/min using a 5–10 μl Hamilton syringe (Hamilton, NV, USA). Bay 11-7085 (an NF-κB inhibitor; 10 μM, Sigma-Aldrich), indomethacin (a COX inhibitor; 10 mg/kg, Sigma-Aldrich) and SHU9119 (an MC3/4R antagonist; 1 nM, Phoenix Pharmaceuticals, Burlingame, CA, USA) were injected 1 h prior to Pam3 injection. After injection of Pam3, animals were allowed ad libitum access to food, and their food intake, body weight, body temperature, and locomotor activity were measured. Minocyline (an inhibitor of microglial activation; 10 μg/2 μl/day, Sigma-Aldrich) was dissolved in 0.9% saline and mice were injected icv with minocycline or saline once a day for 3 consecutive days. On the fourth day, mice were injected icv with saline or Pam3CSK4. Food intake and body weight were measured at 24 h after injection. Microglia depletion was performed by icv administration of liposome-encapsulated clodronate (Clo-lip, 50 μg/7 μl, FormuMax, Sunnyvale, CA, USA). Mice were injected icv with Clo-lip or liposome-encapsulated PBS (PBS-lip) twice a week for 2 weeks. Mice were then injected icv with saline or Pam3CSK4. Food intake and body weight were measured at 24 h after injection of Pam3CSK4.

### Measurement of body temperature and locomotor activity

Body temperature and locomotor activity were measured using biotelemetry transmitters (Mini-Mitter, Bend, OR, USA). Animals were anesthetized with tribromoethanol and the telemetry transmitter was implanted in the peritoneal cavity 1 week before the experiment. After implantation, the layer of the abdominal wall was sutured and the skin incision closed with staples. The output (frequency in Hz) was monitored by a receiver (model RA 1000; Mini-Mitter) placed under each cage. A data acquisition system (Vital View, Mini-Mitter) was used for automatic control of data collection and analysis. Body temperature was recorded at 10-min intervals. Change in locomotor activity was detected as change in the position of the implanted transmitter over the receiver board, which resulted in a change in the signal strength and was recorded as a pulse of activity. Activity pulses were counted every 10 min and average values were calculated after 12 h.

### Western blot analysis

Mediobasal hypothalamus (MBH) samples were homogenized in tissue protein extract reagent (Pierce Chemical Co., Rockford, IL, USA) containing a protease inhibitor cocktail (Roche, Basel, Switzerland; 1 mmol/L phenylmethylsulfonyl fluoride, 10 μg/ml leupeptin, and 3 mM aprotinin) and 1 mM sodium orthovanadate (pH 6.8). Protein samples (15 μg) were resuspended in a reducing sample buffer, separated by SDS-polyacrylamide gel electrophoresis, and blotted onto a polyvinylidenedifluoride (PVDF) membrane (Millipore, Billerica, MA, USA) by electrophoretic transfer. The membrane was incubated overnight at 4 °C with rabbit anti-phosphorylated p65 (Ser-536) antibody (1:1,000 dilution; Cell Signaling Technology, Danvers, MA, USA) and rabbit anti-p65 antibody (1:1,000 dilution; Santa Cruz Biotechnology, Santa Cruz, CA, USA), followed by incubation with horseradish peroxidase-conjugated goat anti-rabbit antibody (1:4,000 dilution; Santa Cruz Biotechnology) overnight at 4 °C. Immunoreactivity was detected using an enhanced chemiluminescence kit (Amersham Biosciences, Hammersmith, UK).

### Immunohistochemistry (IHC)

Mice were deeply anesthetized with tribromoethanol and transcardially perfused with ice-cold 0.9% saline containing heparin (10 mg/L), followed by fresh fixative of 4% paraformaldehyde in phosphate buffer (PB, 0.1 M, pH 7.4). Brains were dissected out and post-fixed overnight in the same fixative. After washing several times in cold PB (0.1 M), coronal brain sections (50 μm thickness) were obtained using a vibratome (VT1000P; Leica Microsystems, Wetzlar, Germany) and sections containing the hypothalamic arcuate nucleus (Arc) were selected under a stereo microscope (Stemi DV4; Carl Zeiss Microimaging Inc., NY, USA). Brain sections were washed several times in PB and incubated with 1% hydrogen peroxide (H2O2) in PB for 20 min to block endogenous peroxidase activity. After washing in PB several times, the sections were preincubated with 0.2% Triton X-100 (Sigma-Aldrich) in PB for 30 min. After further washing in PB, the sections were incubated with primary antibodies as follows: mouse anti-TLR2 antibody (1:1,000 dilution overnight at RT; Biolegend, San Diego, CA, USA), rat anti-TLR2 antibody (1:1,000 dilution overnight at RT; eBioscience, San Diego, CA, USA), rabbit anti-Iba-1 antibody (1:3,000 dilution overnight at RT; Wako, Osaka, Japan), mouse or rabbit anti-GFAP antibody (1:3,000 dilution overnight at RT; Sigma-Aldrich), mouse anti-NeuN antibody (1:1,000 dilution overnight at RT; Millipore), rabbit anti-c-Fos antibody (1:2,000 dilution overnight at RT; Millipore), mouse anti-GAD67 antibody (1:1,000 dilution overnight at RT; Synaptic System, Goettingen, Germany), mouse anti-vGlut2 antibody (1:1,000 dilution overnight at 4 °C; Millipore), and sheep anti-α-MSH antibody (1:10,000 dilution overnight at 4 °C; Millipore). For diaminobenzidine (DAB)-based Iba-1 IHC, sections were extensively washed and incubated in biotinylated anti-rabbit secondary antibody, ABC reagent (Vector Laboratories, Burlingame, CA, USA) and DAB substrate (Vector Laboratories). For immunofluorescence, sections were incubated with combinations of Alexa Fluor 488-, Alexa Fluor 594-, or Alexa Fluor 647-labeled anti-rabbit, anti-sheep, or anti-mouse secondary antibody (1:500 dilution for 1 h at RT; Life Technologies, Carlsbad, CA, USA). Finally, the slice sections were mounted on glass slides and coverslipped with a drop of mounting medium (Dako North America Inc., Carpinteria, CA, USA). The coverslip was sealed with nail polish to prevent drying and movement under the microscope. All slice sections were stored in dark at 4 °C.

### IHC image capture and analyses

Images were recorded by confocal laser-scanning microscopy (TCS SP5; Leica Microsystems, Wetzlar, Germany) and fluorescence microscopy (Axioplan2 Imaging; Carl Zeiss Microimaging Inc.). For all IHC analyses, the slice sections were anatomically matched with the mouse brain atlas^50^ (Arc: between −1.46 and −1.82 mm from bregma, PVN: between −0.82 and −1.06 mm from bregma). Both sides of the bilateral brain region (Arc and PVN) were analyzed for two brain sections per mouse. The number of immunostained cells was counted manually using ImageJ 1.47 v software (National Institutes of Health, Bethesda, MD; http://rsbweb.nih.gov/ij/) by an unbiased observer. Mean microglia staining intensity was measured in a region of interest (ROI) approximating the area of the Arc using ImageJ analysis software, following the on-line tutorials and examples for area measurements and particle counting (http://imagej.nih.gov/ij/docs/pdfs/examples.pdf). Polygonal ROI within an image was manually selected with the mouse brain atlas for Arc. The images were converted to 8-bit images and threshold was applied. The images were binarized to separate the Iba-1-positive cells from the background. The relative intensity was then analyzed for each image by dividing Iba-1 intensity of Pam3CSK4 by that from control group.

To assess the effect of Pam3CSK4-induced microglia activation on the POMC neurons, double (GFP-labeled POMC neuron and Iba-1 positive microglia) or triple-labeled (GFP-labeled POMC neuron, Iba-1 positive microglia and GAD67 or vGLUT2) Arc sections were analyzed. In the Z-stack mode, images were repeatedly acquired in different focus positions for the analysis of individual POMC neuronal cell body. In double-labeled Arc sections, the number of POMC neurons in contact with microglia was calculated as a percentage of the total number of POMC neurons in the microphotograph. The area occupied by Iba-1 positive microglia onto the surface of POMC soma was then measured by using ImageJ analysis software. The percentage of area occupied by Iba-1 positive microglia onto the surface of POMC soma was calculated by dividing the length of Iba-1 positive microglia around the POMC neuron by the length of POMC neuronal circumference. In triple-labeled Arc sections, percentage of the GABAergic innervation or the glutamatergic innervation onto POMC neuron was calculated by dividing the length of GAD67 or vGLUT2 punctate signals surrounding POMC neuronal cell bodies by the length of POMC neuronal circumference in contact with microglia or separated from microglia, based on the previous report^24^. Finally, data were collected for correlation analysis between synaptic inputs onto the POMC neurons and surface area of POMC soma occupied by microglia.

### RNA isolation and quantitative (q) real-time polymerase chain reaction (qPCR)

Animals were sacrificed at different time points after injection of either vehicle or Pam3. The brain was dissected out and mediobasal hypothalamus (MBH) samples were collected under a stereo microscope according to the brain atlas. To collect MBH samples containing the Arc, we cut a slice of two millimeter thick of MBH by using a mouse brain matrix for appropriate regions of the brain and preventing differences in tissue weight (approximately 7~8 mg). Total RNA was extracted by homogenizing tissues in TRIzol reagent (Invitrogen, Carlsbad, CA, USA) according to the protocol supplied by the manufacturer. After homogenization, chloroform (Sigma-Aldrich) was added and the tubes were vortexed vigorously, followed by incubation at room temperature (RT) for 5 min and centrifugation at 14,000 rpm for 20 min at 4 °C. The supernatant containing RNA was transferred to a new tube and an equal volume of isopropanol was added. The aqueous phase was recovered and centrifuged at 14,000 RPM for 20 min at 4 °C. Pellets were washed with 70% ethanol and resuspended in diethylpyrocarbonate (DEPC)-treated water. RNA purity and concentration were determined using a NanoDrop ND-1000 Spectrophotometer (NanoDrop Technologies, Wilmington, DE, USA) with absorbance at 260 and 280 nm. cDNA was synthetized using 1 μg RNA with QIAGEN Whole Transcriptome Kit (#207043, Qiagen Inc., Valencia, CA, USA). qPCR experiment was performed by SYBR Green PCR Master Mix (#RT501M, Enzynomics, Seoul, Korea) using LightCycler 480 Real-Time PCR System (Roche Diagnostics, Mannheim, Germany). Data were normalized to the housekeeping gene glyceraldehyde 3-phosphate dehydrogenase (GAPDH). The 2(-Delta Delta C(t)) method was used to analyze the relative quantification of gene expression^51^. Primer sequences and amplicon sizes for real-time qPCR are shown in Table 1.

### Statistical Analysis

All data are expressed as mean ± s.e.m using GraphPad Prism 5 software (GraphPad, San Diego, CA, USA). The statistical significance between two groups was analyzed by unpaired two-tailed Student’s t-test. Two-way ANOVA analysis was used to compare two treatment groups and two genotypes. A Pearson correlation coefficient (r with p value) was calculated to indicate relationship between the GABAergic or glutamatergic innervation onto POMC neurons and the area occupied by microglia onto the POMC neurons, and linear regression analysis was used to evaluate the statistical significance.

## Additional Information

**How to cite this article**: Jin, S. *et al*. Hypothalamic TLR2 triggers sickness behavior via a microglia-neuronal axis. *Sci. Rep.* **6**, 29424; doi: 10.1038/srep29424 (2016).

## Supplementary Material

Supplementary Information

## Figures and Tables

**Table 1:** Sequences of primers used for real-time quantitative PCR.

Gene	Primer sequence (5′→3′)	Accession number	No. of cycles	Amplicon length (bp)
TLR2	GCGAGTGGTGCAAGTACGAA TGGGCTCCAGCAAAACAAGG	NM 011905.3	45	90
MyD88	CTGCTACTGCCCCAACGATA ACGGTCGGACACACACAACT	NM 010851.2	45	94
TNF-α	TAGCCCACGTCGTAGCAAAC CTTTGAGATCCATGCCGTTG	NM 013693.2	45	96
IL-1β	GTTGACGGACCCCAAAAGAT GATGTGCTGCTGCGAGATTT	NM 008361.3	45	96
COX-2	ACCCTCCTCACATCCCTGAG AAGCCAGATGGTGGCATACA	NM 011198.3	45	95
mPGES-1	ATCAAGATGTACGCGGTGGC GAGGAAATGTATCCAGGCCA	AB 041997.1	45	95
GAPDH	TGCAGTGGCAAAGTGGAGAT TTTGCCGTGAGTGGAGTCAT	NM 008084.2	45	96
