# Supplementary material for: Hypothalamic TLR2 triggers sickness behavior via a microglia-neuronal axis
Source: Sci Rep. 2016 Jul 12;6:29424. doi: 10.1038/srep29424 (PMC4942617; doi:10.1038/srep29424)

**Hypothalamic TLR2 triggers sickness behavior via a microglia-neuronal axis**

Sungho Jin[1,]*, Jae Geun Kim[2, 3,]*, Jeong Woo Park[1], Marco Koch[3, 4], Tamas L. Horvath[3] and Byung Ju Lee[1]

[1]Department of Biological Sciences, University of Ulsan, Ulsan, 680-749, Republic of Korea; [2]Division of Life Sciences, College of Life Sciences and Bioengineering, Incheon National University, Incheon, 406-772, Republic of Korea, [3]Program in Integrative Cell Signaling and Neurobiology of Metabolism, Section of Comparative Medicine, Yale University School of Medicine, New Haven, CT 06520, USA; [4]Institute of Anatomy, University of Leipzig, 04103 Leipzig, Germany.

***** These authors contributed equally to this work. Correspondence and requests for materials should be addressed to T.L.H. or B.J.L. (e-mail: tamas.horvath@yale.edu; bjlee@ulsan.ac.kr)

## Supplementary Information

## Supplementary Figure Legends

**Supplementary Figure S1.** Pam3CSK4-induced activation of microglia was absent in the hypothalamic arcuate nucleus (Arc) of TLR2 KO mice. Representative images show immunosignals of Iba-1 in the hypothalamic arcuate nucleus (Arc). Intracerebroventricular injection of Pam3CSK4 (Pam3) induced activation of microglia in the Arc of wild-type (WT) mice, but not in that of *TLR2* KO mice. CTL = control. Scale bar = 100 µm (20 µm for higher magnification view in inset).

**Supplementary Figure S2.** Effect of Pam3CSK4 on the morphological changes of microglia in the hypothalamus, hippocampus, cortex and circumventricular organs (CVOs). (A,B) Representative images showing immunosignals of Iba-1-positive microglia in the mouse hippocampus and cortex reveal that Pam3-induced central stimulation of TLR2 signaling did not cause morphological change of microglia in these brain regions. (C,D) Representative images show immunosignals of Iba-1-positive microglia in the CVOs, including area postrema (AP) (C) and organum vasculosum of the lamina terminalis (OVLT) (D). Scale bar = 100 µm (20 µm for higher magnification view in inset). CTL = vehicle treated control group. Pam3 = Pam3CSK4 treated group.

**Supplementary Figure S3.** Microglia play a role in Pam3CSK4-induced anorexia and body weight loss. (A) Representative images (3–6 mice analyzed) show immunosignals of Iba-1 in the hypothalamic Arc. Scale bar = 100 μm. CTL = vehicle treated control group. Pam3 = Pam3CSK4 treated group. PBS-lip = liposome-encapsulated PBS treated group. Clo-lip = liposome-encapsulated clodronate treated group. (B) Clo-lip significantly reduced the number of Iba-1-positive microglia in the Arc (CTL, n = 12 sections/6 mice; Pam3, n = 12 sections/6 mice; Clo-lip, 6 sections/3 mice; Clo-lip + Pam3, n = 8 sections/4 mice; ***P < 0.0001 versus CTL mice injected with PBS-lip by unpaired two-tailed Student's t-tests; NS, not significant). (C,D) Pam3-induced anorexia (C) and weight loss (D) were significantly attenuated by administration of Clo-lip prior to icv Pam3 injection. (CTL, n = 4 mice; Pam3, n = 4; Clo-lip, n = 4; Clo-lip + Pam3, n = 4; *P < 0.05, **P < 0.01, ***P < 0.001 by two-way ANOVA). All data are presented as mean ± s.e.m.

**Supplementary Figure S4.** TLR2-induced changes in mRNA expression of genes involved in inflammatory processes in the hypothalamus. (A) Real-time qPCR analysis revealed Pam3CSK4 (Pam3)-induced changes in mRNA expression of *TLR2*, *IL-1β* and *TNF-α* in the hypothalamus and cortex (n = 6 values/2 mice; **P < 0.01, ***P < 0.001 by two-way ANOVA; NS, not significant). (B,C) To further confirm the effect of TLR2 activation on hypothalamic inflammation, real-time qPCR was performed using mRNA samples from the hypothalami of *TLR2* KO (B, n = 9 values/3 mice; ***P < 0.001 by two-way ANOVA; NS, not significant) and *MyD88* KO (C, n = 6 values/2 mice; ***P < 0.001 by two-way ANOVA; NS, not significant) mice 3 h after icv administration of Pam3. The Pam3-induced increases in mRNA expression of genes associated with inflammatory processes were almost completely suppressed in the *TLR2* and *MyD88* KO mice. CTL = control. WT = wild-type mice.

**Supplementary Figure S5.** Central activation of TLR2 increases the number of c-Fos signals in the hypothalamic arcuate nucleus (Arc). To determine the effect of Pam3CSK4 (Pam3) treatment on neuronal activity of the Arc, immunohistochemical observation of c-Fos activity was performed using sections from mice after icv treatment with Pam3, in comparison with control (CTL). Representative images (A) and calculated data (B) reveal that the number of c-Fos-positive cells in the Arc increased by 3 h after icv administration of Pam3 (CTL, n = 8 sections/4 mice; Pam3, n = 6 sections/3 mice; ***P < 0.001 by unpaired two-tailed Student's t-tests). All data are presented as mean ± s.e.m. Scale bar = 100 μm.

**Supplementary Figure S6.** Simplified diagram showing TLR2-induced microglial activation that stimulates POMC neuronal activity and induces anorexia. TLR2 signaling contributes to microglial activation and induces inflammatory reactions via NF-κB and COX pathways in the hypothalamic Arc. The activated microglia, characterized by change in their morphology, regulate synaptic input organization onto the POMC neurons through increased contact area onto the surface of POMC soma. Consequently, inhibitory GABAergic inputs decrease and excitatory glutamatergic inputs increase on the surface of POMC soma contacted by microglia. These synaptic changes result in activation of the POMC neurons, which causes anorexia via α-MSH binding to melanocortin (MC) 4 receptors on the targets of POMC neurons such as neurons in the paraventricular nucleus (PVN).

**Supplementary Figure S1**. Pam3CSK4-induced activation of microglia was absent in the hypothalamic arcuate nucleus (Arc) of TLR2 KO mice

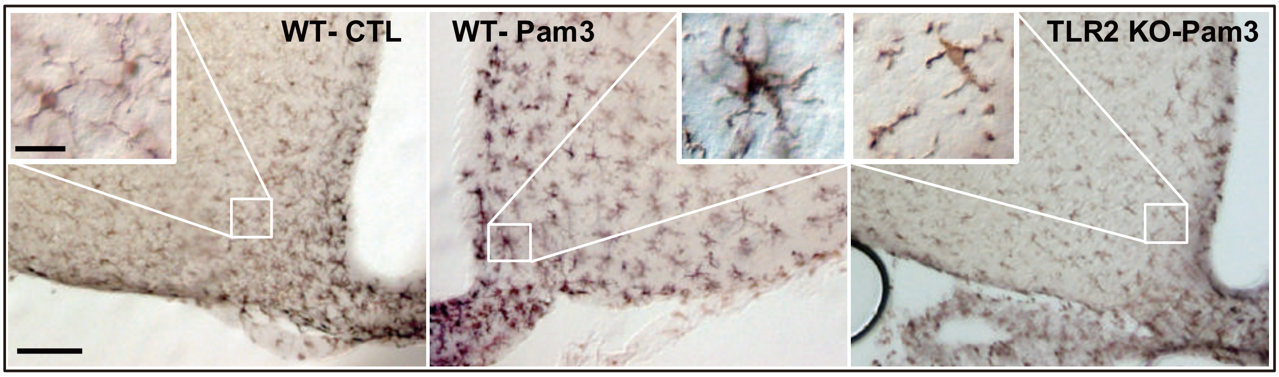

**Supplementary Figure S2**. Effect of Pam3CSK4 on the morphological changes of microglia in the hypothalamus, hippocampus, cortex and circumventricular organs (CVOs)

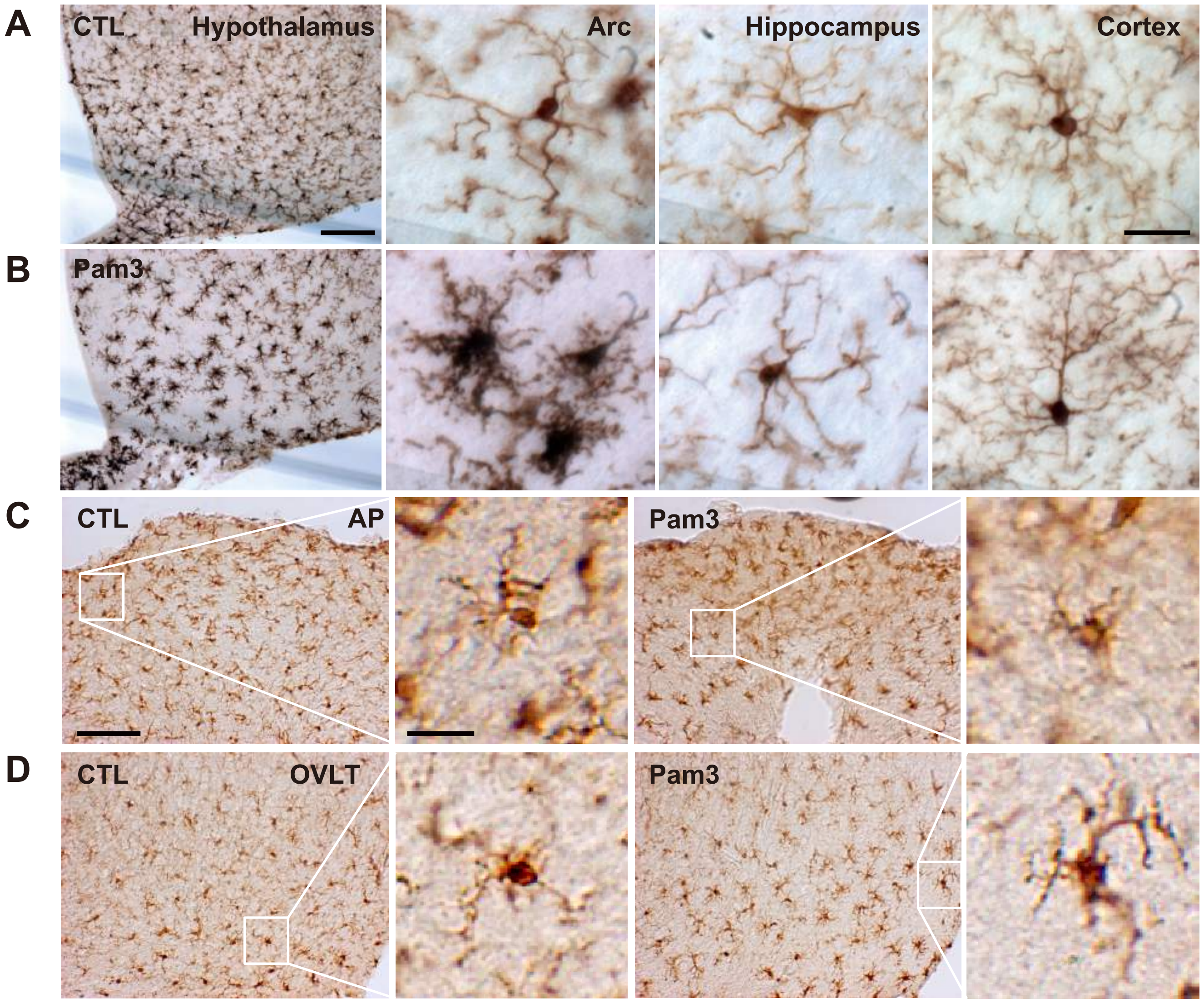

**Supplementary Figure S3**. Microglia play a role in Pam3CSK4-induced anorexia and body weight loss

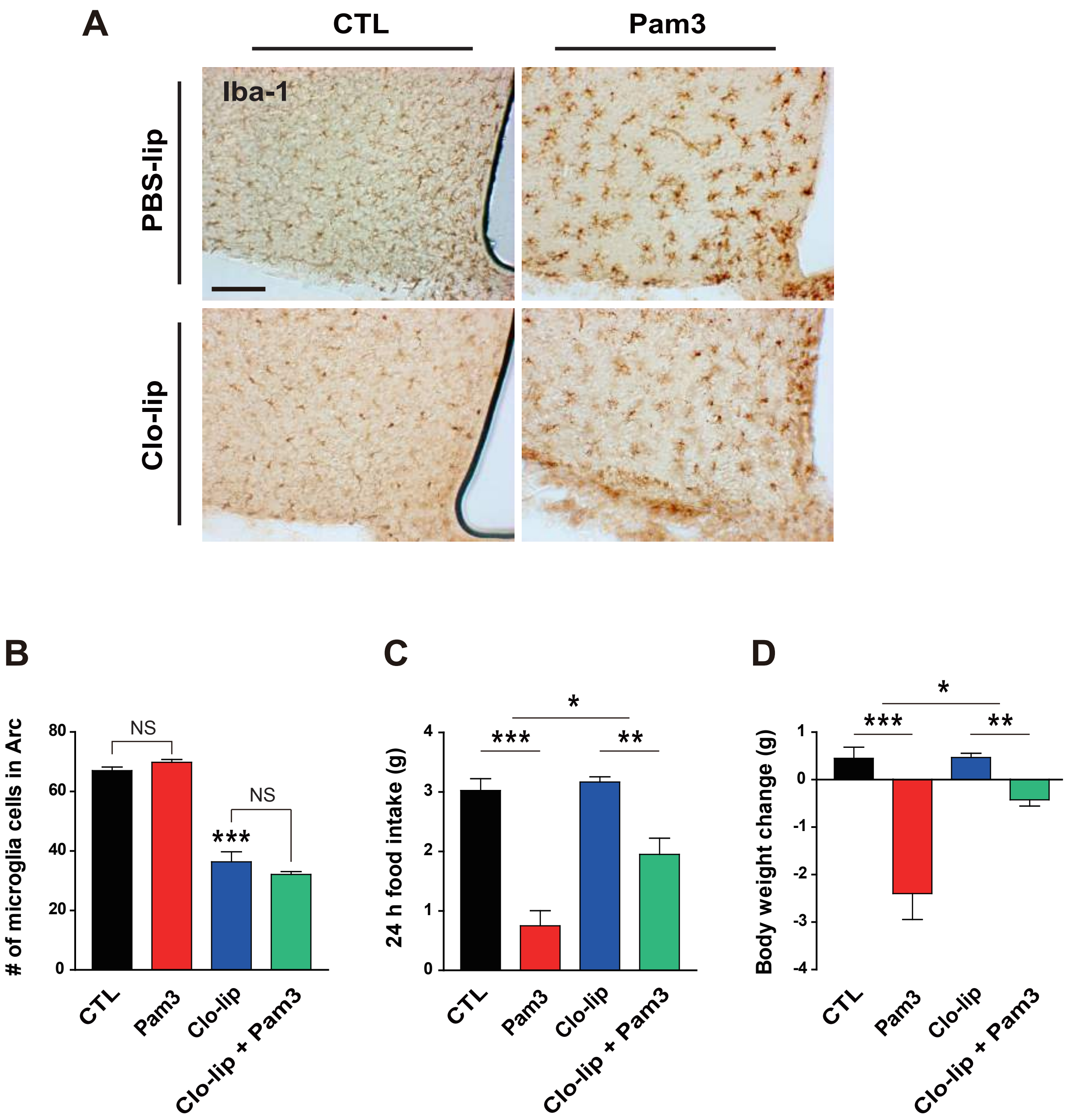

**Supplementary Figure S4**. TLR2-induced changes in mRNA expression of genes involved in inflammatory processes in the hypothalamus

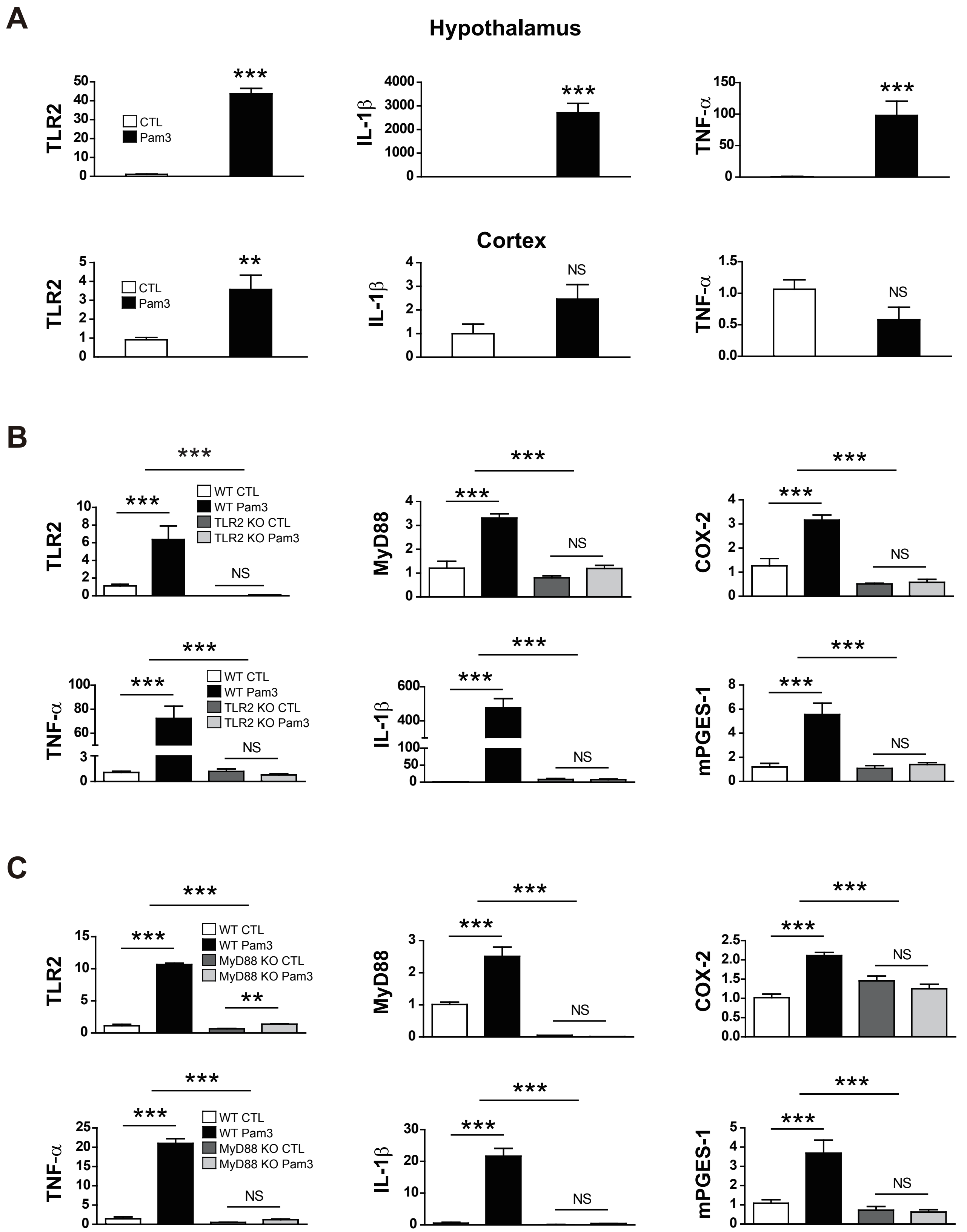

**Supplementary Figure S5**. Central activation of TLR2 increases the number of c-Fos signals in the hypothalamic arcuate nucleus (Arc)

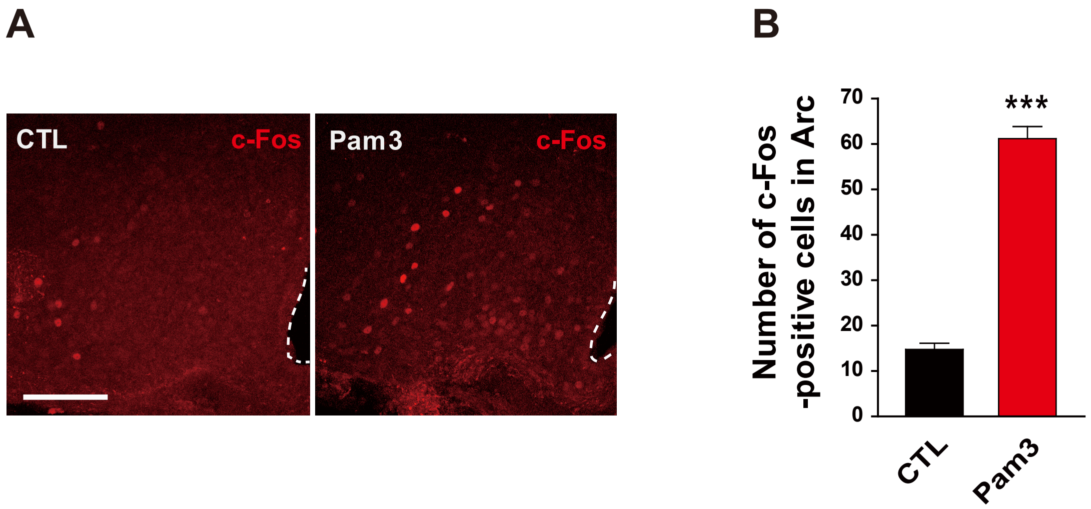

**Supplementary Figure S6**. Simplified diagram showing TLR2-induced microglial activation that stimulates POMC neuronal activity and induces anorexia

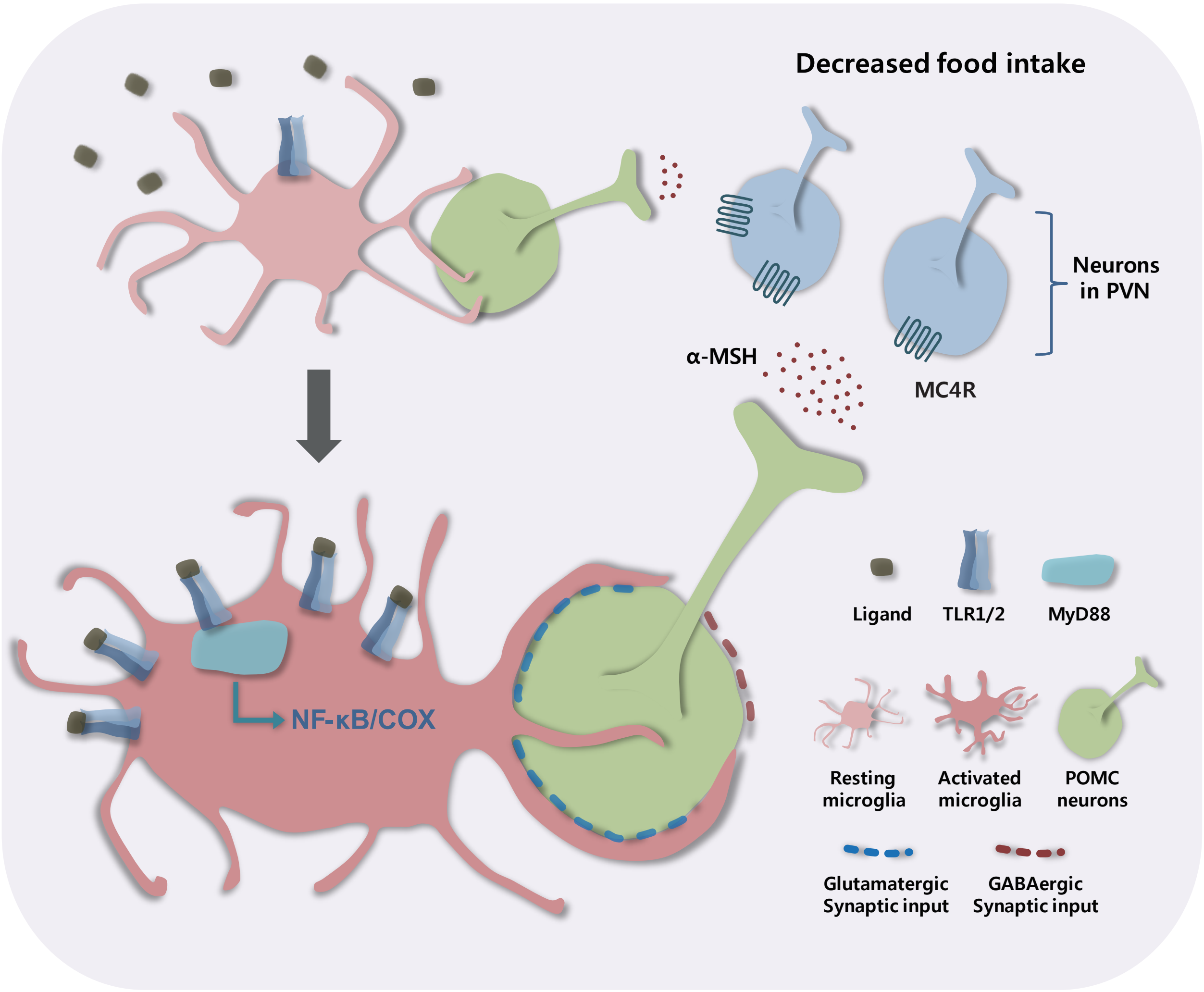

Supplement: Supplementary Information [file srep29424-s1.pdf]
